# Antimicrobial Potential of Bacteria Associated with Marine Sea Slugs from North Sulawesi, Indonesia

**DOI:** 10.3389/fmicb.2017.01092

**Published:** 2017-06-14

**Authors:** Nils Böhringer, Katja M. Fisch, Dorothee Schillo, Robert Bara, Cora Hertzer, Fabian Grein, Jan-Hendrik Eisenbarth, Fontje Kaligis, Tanja Schneider, Heike Wägele, Gabriele M. König, Till F. Schäberle

**Affiliations:** ^1^Institute for Pharmaceutical Biology, University of BonnBonn, Germany; ^2^Institute for Insect Biotechnology, Justus Liebig University of GiessenGiessen, Germany; ^3^Centre of Molecular Biodiversity, Zoological Research Museum Alexander KoenigBonn, Germany; ^4^Faculty of Fisheries and Marine Science, Sam Ratulangi UniversityManado, Indonesia; ^5^German Center for Infection Research Partner Site Bonn-CologneBonn, Germany; ^6^Institute of Medical Microbiology, Immunology and Parasitology – Pharmaceutical Microbiology Section, University of BonnBonn, Germany

**Keywords:** antibiotics, microbiome, natural product, NRPS, marine Heterobranchia, Nudibranchia, PKS

## Abstract

Nudibranchia, marine soft-bodied organisms, developed, due to the absence of a protective shell, different strategies to protect themselves against putative predators and fouling organisms. One strategy is to use chemical weapons to distract predators, as well as pathogenic microorganisms. Hence, these gastropods take advantage of the incorporation of chemical molecules. Thereby the original source of these natural products varies; it might be the food source, *de novo* synthesis from the sea slug, or biosynthesis by associated bacteria. These bioactive molecules applied by the slugs can become important drug leads for future medicinal drugs. To test the potential of the associated bacteria, the latter were isolated from their hosts, brought into culture and extracts were prepared and tested for antimicrobial activities. From 49 isolated bacterial strains 35 showed antibiotic activity. The most promising extracts were chosen for further testing against relevant pathogens. In that way three strains showing activity against methicillin resistant *Staphylococcus aureus* and one strain with activity against enterohemorrhagic *Escherichia coli*, respectively, were identified. The obtained results indicate that the sea slug associated microbiome is a promising source for bacterial strains, which hold the potential for the biotechnological production of antibiotics.

## Introduction

The increased life expectancy of humans is the great success story of antibiotics. However, nowadays antibiotic-resistant bacteria are on the rise, a circumstance regarded as one of the biggest threats to human health (Schäberle and Hack, 2014; WHO Library Cataloguing-in-Publication Data, 2014). Hence, new antibiotic drugs are urgently needed. To fulfill this task, classical screening methods for new bioactive compounds were revived in recent years. A promising strategy to identify novel biologically active compounds is to explore new habitats. Compared to the terrestrial environment, the oceans are still under-investigated, even though they make up two third of the planet’s surface, and harbor high biodiversity with an immanent high potential for discovery of new metabolites (Donia and Hamann, 2003). The rationale underlying the idea of targeting the defined biological niche of marine sea slugs, their food source and associated microorganisms is the highly competitive nature of this environment. The slugs must protect themselves against various predators, e.g., fish and crabs, and against settlement of pathogenic microorganisms on their epidermis. To reach this, chemical defense medated by natural products plays a major role. It was already shown that the slugs incorporate and enrich bioactive metabolites from their food source (Bogdanov et al., 2014, 2016). However, it has to be kept in mind that compounds present in the food source may be rather produced by associated microorganisms, e.g., bacteria. The antitumor agent dolastatin 10 was first described from the anaspidean *Dolabella auricularia* (Anaspidea). This compound was thought to be sequestered from algae, the food source of the heterobranchs Sacoglossa and Anaspidea. However, more recent findings of dolastatin 10 in cyanobacteria impose an origin in bacteria associated with the algal food (Luesch et al., 2001; Cimino and Gavagnin, 2006; Gerwick and Fenner, 2013). A similar relationship between bacteria and higher organisms can also be expected for many compounds originally described from sponges (Mehbub et al., 2014; Wilson et al., 2014).

In this article, a culture-dependent approach to analyze the potential of the slug-associated microbiome is described. The isolated bacteria were tested for the presence of polyketide synthases (PKSs) and non-ribosomal peptide synthetases (NRPSs) coding genes, since a majority of pharmaceutical interesting compounds is produced by these biosynthetic pathways. Further, the available extracts from axenic bacterial cultures were tested for antimicrobial activity against relevant pathogens.

## Materials and Methods

### Environmental Samples

**FIGURE 1:**
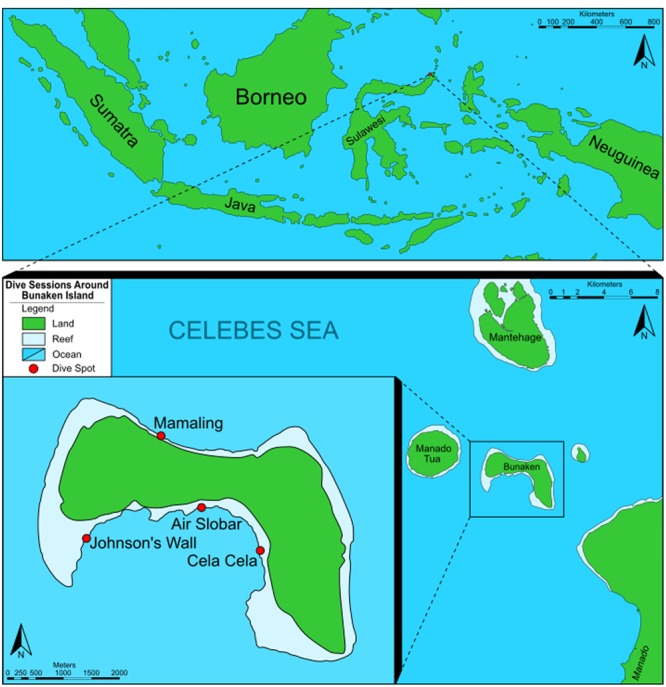
Map of the sampling sites around Bunaken Island, Bay of Manado, Sulawesi, Indonesia.

Sampling was performed at the Bunaken National Park in August 2015, located near Manado, North Sulawesi, Indonesia. In total, 550 samples for phylogenetic, chemical and microbiological studies were obtained under the framework of the “Indobio” Biodiversity and Health project from different sites around the island by snorkeling and scuba diving (**Figure 1**). Further information about the samples will be published elsewhere. The samples were either directly processed, or cooled until further processing in the lab.

### Identification of Sea Slugs and Sponges

**FIGURE 2:**
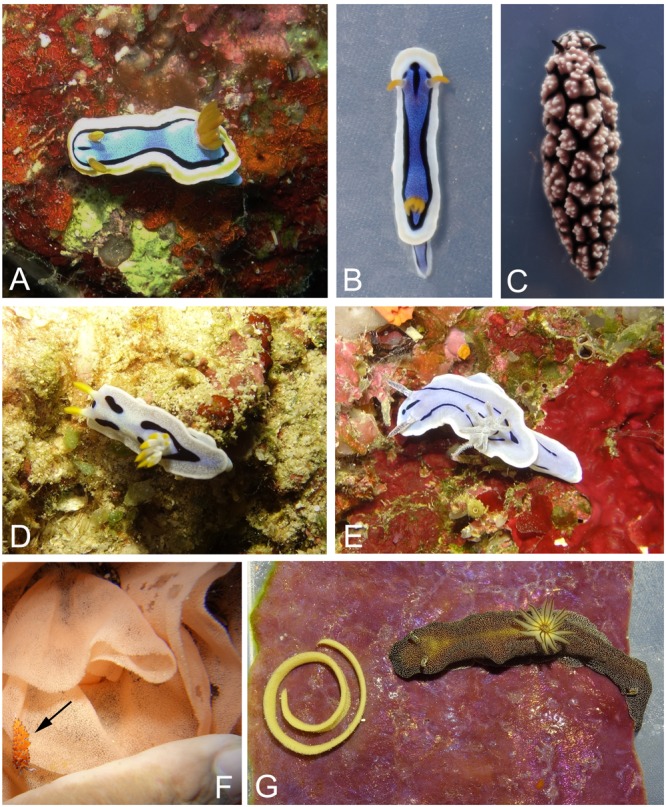
Higher organisms used in this study: **(A)** *Chromodoris annae* (Chan15Bu-2) in its natural environment. **(B)** *C. annae* (Chsp30_15Bu-4) from same locality but with different coloration. **(C)** *Phyllidiella* cf *pustulosa* (Phpu15Bu-1). **(D)** *C. dianae* (Chdi15Bu-55) in its natural environment. **(E)** *C. willani* (Chwi15Bu-2) in its natural environment. **(F)** *Hexabranchus sanguineus* egg mass (Hesa15Bu-2) in its natural environment and with a nudibranch, *Favorinus tsuruganus* feeding on the eggs (arrow). **(G)** *Dorioprismatica stellata* (Glst15Bu-1) with its egg mass (Glst15Bu-1E) on the typical sponge *Phyllospongia* cf *lamellosa* (Glst15Bu-1P).

Preliminary identification of slugs and egg masses was performed based on Gosliner et al. (2008, 2015) (**Figure 2**), identification of sponges based on Atlas of living Australia^1^. For barcoding, a small portion of the sampled slugs, egg masses and sponges was stored in 96% EtOH and the DNA was isolated using QIAgen DNeasy Blood and Tissue-Kit (Qiagen, Hilden, Germany). Partial 16S rDNA sequences and partial CO1 gene sequences were amplified using 16Sar-5′ and 16Sbr-3′ primers (Palumbi, 1996) and LCO1490-JJ and HCO2198-JJ primers (Astrin and Stüben, 2008) and sent to Macrogen Inc. (Seoul, Korea). The specimens’ identities were determined by subsequent blastn analysis of the obtained sequences.

### Isolation of Bacteria

Prior to the isolation of bacteria, the surface of the samples (in the following the identification code of each sample is used; further information can be found via the internet portal of Diversity Workbench within the module DiversityCollection^2^. Chan15Bu-2 (*Chromodoris annae*), Chdi15Bu-3, Chdi15Bu-55 and Chdi15Bu-38 (*C. dianae*), Chsp3015Bu-4 (*Chromodoris* sp. 30), Chwi15Bu-2 (*C. willani*), Chan15Bu-11E (*C. annae* egg mass), Glst15Bu-1 (*Doriprismatica stellata*), Glst15Bu-1E (*D. stellata* egg mass), Glst15Bu-1P (*D. stellata* sponge), Hesa15Bu-1 and Hesa15Bu-2 (*Hexabranchus sanguineus* egg mass) and Phpu15Bu-1 (*Phyllidiella* cf *pustulosa*) were washed with sterile deionized H_2_O, disinfected with 70% ethanol and washed again with sterile deionized H_2_O (Milli-Q, Merck, Germany) to remove contaminations from sampling and documentation. The samples were frozen in liquid nitrogen and homogenized using a Potter S Homogenizer (Sartorius, Germany). The homogenisate was brought onto Marine Broth agar plates (37.9 g/L Marine Broth (Difco), 15 g/L agar) or ISP2 agar plates containing 2% NaCl (4 g/L yeast extract, 10 g/L malt extract, 4 g/L glucose, 20 g/L NaCl, 15 g/L agar) using an inoculation loop and the 13 streaks technique to obtain axenic colonies. Axenic colonies were transferred to new plates. For cryo-conservation, axenic cultures were grown in the respective liquid medium, mixed 50:50 with sterile glycerol and stored at -80°C.

### Generation of Extracts

Bacteria were grown in 30 mL of the respective medium. Therefrom, 25 mL were transferred to a 50 mL reaction tube, 25 mL ethyl acetate were added and the tube was shaken thoroughly. The organic phase was carefully removed with a pipette and evaporated. The extracts were dissolved to 1 mg/mL in methanol for HR-LCMS and to 10 mg/mL in DMSO for antibiotic testing.

### Antibacterial Assays

For antibacterial assays, test strains were grown in 200 μL cultures in flat bottom 96 well plates at room temperature. The initial OD_600_ was set to 0.1 and the extracts were added to yield a final concentration of 0.5 μg/μL per culture for the first well and subsequently diluted 1:1 in the following wells to the lowest concentration of 4 ng/μL. 0.5 μg/μL ampicillin served as positive control, pure DMSO as negative vehicle control. The tests were evaluated when the negative control reached an OD_600_ of 1.0. Extracts were tested against *Arthrobacter psychrolactophilus* and *Escherichia coli* XL1Blue. For the preparation of *Staphylococcus aureus* COL and *E. coli* O-19592 assay plates, cation adjusted Müller Hinton agar was autoclaved and allowed to cool down to 50–55°C. 10^7^ cells/ml of the test strains were seeded into the agar and after solidification, 3 μl of the ethyl acetate extracts were spotted onto the plates. Ampicillin (0.1 μg/ml) and ethyl acetate served as positive and negative controls, respectively. Plates were incubated at 37°C for 18 h and checked for inhibition zones.

### Detection of PKS and NRPS Biosynthetic Genes

Genomic DNA of the different bacterial strains was isolated using the GenElute Bacterial Genomic DNA Kit (Sigma) according to the manufacturer protocol. The genomic DNA was subjected to several PCR screening rounds using degenerated primers (see Supplementary Table 1). Primers were based on literature, or designed based on conserved regions in ketosynthase (KS) domains of PKS and adenylation domains (A-domains) of NRPS. Specific PCR for serine activating A-domains was performed as nested PCR, i.e., the PCR products of a general A-domain PCR with degenerated primers were gel purified and used in a subsequent PCR with AnSerin/JS002 as primer pair. The amplified products of approximately 700bp (KS domains), 700bp (A3–A7 region of A-domains), 1000bp (A2–A8 region of A-domains) and 450bp (serine specific nested PCR) were gel purified and cloned into pGEM-T vector (Promega, Madison, WI, United States), transformed into chemical competent *E. coli* Alpha Silver Select Efficiency (Bioline, Luckenwalde, Germany) and sequenced. Sequences were analyzed using blastx and blastn and closest protein sequences of were analyzed for amino acid specificity using antiSMASH 3.0 (Weber et al., 2015) and submitted to Genbank. The accession numbers for this project are KY671135 – KY671183 (16S rDNA), KY671132 – KY671134 (KS), and KY671184 – KY671199 (A domains).

### Phylogenetic Analysis

The bacterial species were determined by 16S rDNA sequencing. Therefore, genomic DNA was isolated from 2 mL of liquid culture using the GenElute Bacterial Genomic DNA Kit (Sigma) and the 16S rDNA region was amplified by PCR using rD1 and fD1 primers (Weisburg et al., 1991). The resulting amplificate was cloned into pGEM-T vector (Promega, Madison, WI, United States), introduced into *E. coli* Alpha Silver Select Efficiency cells (Bioline, Luckenwalde, Germany). The plasmids were isolated using the PureYield Miniprep kit (Promega, Madison, WI, United States) and the insert was sequenced from both sides using T7 and SP6 primers (GATC, Konstanz, Germany). Vector fragments in the sequencing result were removed using VecScreen-Blast and the full 16S rDNA was assembled. BLASTn search of the 16S rDNA sequences revealed the closest relatives, thereby enabling determination of the species, due to the fact that identity to known type strains was in the range of 98–100%. The phylogenetic tree of all obtained bacterial isolates was created by aligning the 16S rDNA sequences of the respective type strains using MAFFT (Katoh et al., 2002) and constructing the tree using raxML (Stamatakis, 2014).

## Results

### Isolation and Identification of Sea Slug Associated Bacteria

Preliminary identification of the slugs and egg masses were confirmed by barcoding. Metadata of the collected macroorganisms will become available via the internet portal of Diversity Workbench within the module DiversityCollection^3^. 49 bacterial strains were isolated as axenic culture starting from 12 different samples. The highest proportion, of strains (20% each) was isolated from the egg masses Hesa15Bu_1 and Hesa15Bu_2 of *H. sanguineus* whereby only 2% (equates to 1 strain) were isolated from the processed sponge sample. Most of the strains were recovered using Marine Broth standard medium, while 10 strains were recovered by using ISP2 medium optimized for actinobacteria (see Materials and Methods). For a full table of strains and their respective source see Supplementary Table 2.

### Antimicrobial Activity

**FIGURE 3:**
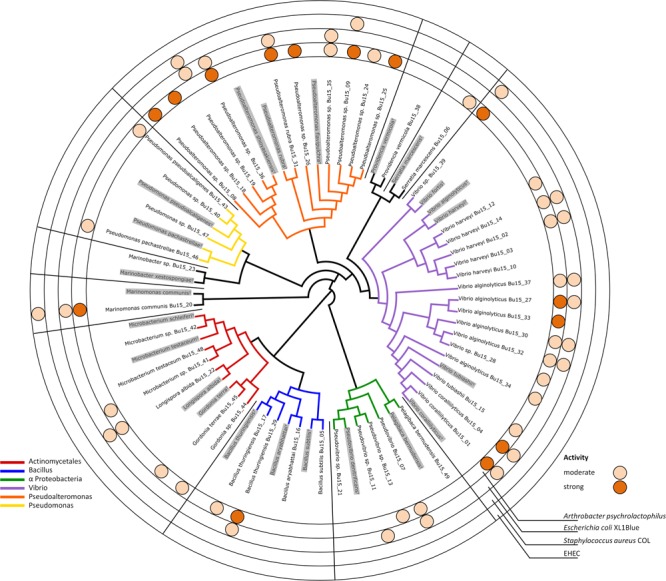
Phylogenetic tree of the isolated axenic bacterial strains and the respective closest type strain (marked in gray). Detected activities toward Gram-positive and Gram-negative test strains, thereunder clinically relevant MRSA and EHEC isolates, are indicated with dark orange (strong activity) and mildly orange (weak activity) circles.

Axenic bacterial strains were cultivated in liquid medium and ethyl acetate crude extracts were prepared. These extracts were first tested against the Gram-positive test strain *Arthrobacter psychrolactophilus* and the Gram-negative test strain *E. coli* XL1Blue. 35 strains were tested positive in this assay. However, to prioritize the samples, the 10 most active extracts were selected for further testing against relevant pathogens, i.e., *S. aureus* COL (MRSA) and *E. coli* O-19592 (EHEC). In this assay, three extracts showed anti-MRSA activity and one extract showed anti-EHEC activity (**Figure 3**). It can be seen that especially isolates of *Pseudoalteromonas* strains showed considerable activity. However, the strain showing activity against the Gram-negative EHEC strain was a *Marinomonas* species.

### Phylogenetic Analysis

For all isolated axenic bacteria the 16S rDNA gene was amplified using rD1 and fD1 primers, designed for the specific and long range amplification of eubacterial 16S rDNA. All isolated strains belong to known genera and show high homology (98–100% identity) to sequences stored in the database. The closest homolog type strain of each isolated strain was included in an alignment to obtain a phylogenetic tree (**Figure 3**).

From the obtained isolates, 35 out of 49 strains (equal to 71%) showed antibacterial activity. All of the 10 isolated *Pseudoalteromonas* strains showed activity, and 7 thereof revealed strong activity against the Gram-positive test strain. Further, two strains also inhibited methicillin resistant *S. aureus* (MRSA). Another bacterial group with a high proportion of strains showing antibacterial effects was the *Vibrio* group. Therein, 14 out of 16 isolated strains showed at least slight activity, 5 strains exhibited strong activity against the Gram-positive test strain, and 1 strain showed anti-MRSA activity.

One isolate even showed activity against enterohemorrhagic *E. coli* (EHEC). This strain was the only isolate from the *Marinomonas* clade.

### Detection of PKS and NRPS Biosynthetic Genes

All 49 strains were tested for the presence of DNA sequences known to be associated with biosynthetic gene clusters (BGCs) contributing to the production of specialized metabolites, i.e., degenerated primers were used to screen the genomic DNA for the presence of NRPS sequences. Primers were adapted from literature to target conserved regions of A-domains. Despite many trials, the number of sequences obtained by this approach was very low, i.e., 10 A-domain sequences (6 different from *Gordonia terrae* Bu15_45, 2 different from *Pseudoalteromonas rubra* Bu15_31, 1 from *Pseudoalteromonas* sp. Bu15_09 and 1 from *Gordonia* sp. Bu15_44). Since oxazole natural compounds are amongst valuable antibiotics, such as streptogramin A, a two-step nested PCR approach for serine specific A-domains was projected by designing a primer between the conserved regions A3 and A7 with specificity to serine incorporating A-domains (AnSerin see Supplementary Table 1). This enabled us to obtain further 6 sequences (1 from *Vibrio coralliilyticus* Bu15_04, 3 different ones from *Pseudoalteromonas* sp. Bu15_09, 1 from *Pseudoalteromonas* sp. Bu15_26 and 1 from *Bacillus thuringiensis* Bu15_29). All except one proved to be partial A-domain sequences with first blastx hits of A-domains predicted to incorporate serine.

*Gordonia* sp. Bu15_44, *P. rubra* Bu15_31, *V. coralliilyticus* Bu15_01 and *Pseudoalteromonas* sp. Bu15_09 were tested for the presence of DNA sequences associated with PKS BGCs, i.e., degenerated primers (see Supplementary Table 1) amplifying partial KS fragments were used. Only *Gordonia* sp. Bu15_44 yielded 3 distinct KS fragments.

## Discussion

The isolation of *Vibrio* and *Pseudoalteromonas* strains from marine sea slugs seemed likely, since these bacteria are known as abundant species in the marine habitat, and many strains were already isolated by using commercially available complex media with a concentration of ions mimicking seawater. In this screening effort, many *Pseudoalteromonas* strains were isolated and all of them showed at least slight antibacterial activity in the tests used. The genus *Pseudoalteromonas* currently consists of 41 species, thereunder 16 are known as producers of antimicrobials. The various compounds isolated to date were recently reviewed (Offret et al., 2016), and reported natural products are alkaloids, polyketides and peptides. The bacteria are frequently associated with macroorganisms and therefrom it can be expected that the chemical molecules fulfill an important role as signaling and/or defense molecules. These bacteria are mostly associated with healthy animals and plants, only few reports exist that these strains act opportunistic or pathogenic (Choudhury et al., 2015). Therefore, a positive effect on the implementation of the microbiome for the macroorganism can be expected, making them a key partner in marine holobionts. The latter is composed of the host and its microbiome. A regulation of the microbiome is desirable for invertebrates, since they have to protect their epidermis from unwanted settlement with pathogenic biofilm members. The analysis of these talented antimicrobial producers as probiotics in aquaculture and the analysis of produced molecules as potential lead structures for medicinal drugs will be subject of future studies. In contrast to the Pseudoalteromonaceae, the dominant marine bacterial family, i.e., Vibrionaceae, consists of 126 species and only 6 of them are known to produce antimicrobials (Mansson et al., 2011). In the here described bioprospecting approach several *Vibrio* strains showed no antimicrobial activity, but one strain possessed anti-MRSA activity. This exemplifies that the pangenome of the Vibrionaceae indeed carries the potential to synthesize antimicrobials, and that this must be evaluated individually for the respective strains. The bacterial strain showing anti-EHEC activity belongs to the genus *Marinomonas*. Members of this genus were isolated from most diverse habitats throughout the ocean. However, up to now they were not noticeable as prolific producers of natural products. *Marinomonas mediterranea* was described as the producer of marinocine, a broad-spectrum antibacterial protein (Lucas-Elio et al., 2005). Further, the natural product Indole-3-carboxaldehyde biosynthesized by *Marinomonas* sp. was recently described as an anti-biofilm agent against *V. cholerae* O1 (Rajalaxmi et al., 2016). The molecule(s) responsible for the here observed activity will be subject of future studies.

The efforts to get members of the sponge microbiome growing *in vitro* is a good example for the plate-anomaly, i.e., the number of bacterial cells which form colonies on an agar plate is much less than bacterial cells present in the sample. Many natural products originally isolated from sponges can be attributed to bacteria as the real producers (Mehbub et al., 2014). However, despite big efforts of the scientific community, it remains most challenging to bring these bacteria into culture (Waters et al., 2014). This might explain why in the here described research only one strain, i.e., *V. alginolyticus* Bu15_37, was isolated starting with sponge material.

An interesting outcome of this study is that a large portion of bacteria with antimicrobial activity was isolated from egg masses. It can be speculated that this points toward a symbiotic relationship between sea slugs and bioactive compounds producing bacteria. Beside the sea slug, which is constantly producing mucus to clean the epidermal surface also the egg masses must be protected, even though the offspring, with a high content of lipids and proteins, represents a highly desirable food source. A capsule membrane and various mucus layers surround these eggs. However, to ensure the stability and protective properties of the whole egg mass for the time necessary before veliger or juveniles hatch, quick degradation through bacteria and other organisms has to be prevented. Thus, it can be assumed that biologically active compounds play an essential role in egg mass conservation. This was shown for egg masses of *H. sanguineus* that is protected by the macrolide kabiramide C with antifouling properties (Matsunaga et al., 1986). In the meantime, more kabiramides, up to kabiramide L were identified (Sirirak et al., 2013). The effect of such natural antifouling agents toward the protection of egg masses is hardly investigated yet, but the finding of bacteria in the distal part of the nidamental glands in the tropical sea slug *Dendrodoris nigra* is highly interesting. *D. nigra* lives in the upper sublittoral of coral reefs, and could provide its eggs with bacterial metabolites before the egg mass is subsequently released from the oviduct (Brodie et al., 1999). Further, bacteria inhabiting the surface of the mucus layers of *Siphonaria* egg masses were identified which inhibit growth of *V. harveyi* (Peters et al., 2011). Investigation of the interrelation between associated bacteria and desired protection of egg masses opens up a topic for future research.

To tackle the threat by antibiotic-resistant bacteria, new treatment options and new bioactive compounds must be identified. The last decades showed that the target-based screening approaches were not as successful as expected. Instead, the “classical” bioactivity-based approach of identifying new sources and the isolation of natural products with the targeted activity is still most rewarding. This is proven by the fact that about 80% of all antimicrobial drugs in current use are natural products or are based on their structures (Newman and Cragg, 2012). To get a hand on novel compounds, so far under-investigated biological niches are now explored. Bioprospecting anaerobic bacteria (Akita et al., 2015), resulted in the isolation of a novel producer strain, enabling the description of the highly active clostrubin (Pidot et al., 2014). In addition, methods were developed to enable *in vitro* growth of so far unculturable bacteria. This approach resulted in the identification of teixobactin, an antibiotic without detectable resistance (Ling et al., 2015). Another approach is reinvestigation of known antibiotic compounds, which were not developed further in the Golden Age of antibiotic research were enough treatment options had been available. A promising example is griselimycin that is reinvestigated with the goal to develop it into a clinical drug (Kling et al., 2015). Hence, it became clear that Nature is still the most promising resource for novel compounds with antibiotical activity. In this context the presence of PKS- and NRPS-coding sequences in the isolated strains provides further evidence that biosynthesis of specialized metabolites with interesting biological activities is encoded in these bacteria. Therefore, the potential of the sea slug associated microbiome for production of natural products harboring the inherent potential to be developed into drug leads must be judged as very high.

## Author Contributions

FK, RB, HW, GK, and TS designed the research and analyzed results. All authors were involved in sampling, and/or performing experiments, and wrote parts of the manuscript prepared by NB and TS. All the authors discussed the results and commented on the manuscript.

## Conflict of Interest Statement

The authors declare that the research was conducted in the absence of any commercial or financial relationships that could be construed as a potential conflict of interest.

## Footnotes

1 https://www.ala.org.au/

2 http://diversityworkbench.net/Portal/DiversityCollection

3 http://diversityworkbench.net/Portal/DiversityCollection
