# Supplementary material for: Antimicrobial Potential of Bacteria Associated with Marine Sea Slugs from North Sulawesi, Indonesia
Source: Front Microbiol. 2017 Jun 14;8:1092. doi: 10.3389/fmicb.2017.01092 (PMC5469899; doi:10.3389/fmicb.2017.01092)
Supplement: Supplementary file 1 [file Data_Sheet_1.DOCX]

Supplementary Material

Antimicrobial Potential of Bacteria Associated with Marine Sea Slugs from North Sulawesi, Indonesia

Nils Böhringer^1,2^, Katja M. Fisch^1,2^, Dorothee Schillo^3^, Robert Bara^4^, Cora Hertzer^1^, Fabian Grein^5,6^, Jan-Hendrik Eisenbarth^3^, Fontje Kaligis^4^, Tanja Schneider^5,6^, Heike Wägele^3^, Gabriele M. König^1,6^, Till F. Schäberle^1,2,6*^

^1^Institute for Pharmaceutical Biology, University of Bonn, Bonn, Germany

^2^Institute for Insect Biotechnology, Justus Liebig University of Giessen, Giessen, Germany

^3^Center of Molecular Biodiversity, Zoological Research Museum Alexander Koenig, Bonn, Germany

^4^Faculty of Fisheries and Marine Science, Sam Ratulangi University, Manado-Indonesia

^5^German Center for Infection Research (DZIF) Partner Site Cologne/Bonn

^6^Institute of Medical Microbiology, Immunology and Parasitology - Pharmaceutical Microbiology Section, University of Bonn, Bonn, Germany

*** Correspondence:**till.f.schaeberle@agrar.uni-giessen.de

# Supplementary Figures and Tables

## Supplementary Tables

**Supplementary Table 1.** Primers used for screening of BGCs

| Primer name | Sequence 5’-> 3’ | specificity | Literature |
| --- | --- | --- | --- |
| JS002=A7Rallg | SASRTCNCCNGTNCGRTASA | A-domain A7 | this study, based on A7R of Ayuso-Sacido, A. and Genilloud, O. (2005) |
| A3F | GCSTACSYSATSTACACSTCSGG | A-domain A3, GC rich | Ayuso-Sacido, A.and Genilloud, O. (2005) |
| A7R | SASGTCVCCSGTSCGGTAS | A-domain A7, GC rich | Ayuso-Sacido, A.and Genilloud, O. (2005) |
| JS007 | AARDSNGGNGSNGSNTAYBNCC | A-domain, A2 | based on degNRPS-1F.i Schirmer et al. 2005 |
| JS008 | CKRWRNCCNCKNANYTTNACYTG | A-domain, A8 | based on degNRPS-4R.i Schirmer et al. 2005 |
| NRPSA-F | GGWCDACHGGHMANCCHAARGG | A-domain A3 | Wu et al. 2011 |
| NRPSA-R | GGCAKCCATYTYGCCARGTCNCCKGT | A-domain A7 | Wu et al. 2011 |
| AnSerin | CAYTTYGTNCCNWSNATGYT | A-domain, serine | this study |
|  |  |  |  |
| KF0001=  KSDPQQFi | MGi GAR GCi HWi SMi ATG GAY CCi CAR CAi MG | general KS-specific primer | based on Fisch et al. 2009 |
| KF0002=  KSHGTGRi | GGR TCi CCi ARi SWi GTi CCi GTi CCR TG | general KS-specific primer | based on Fisch et al. 2009 |
| KF0003=  KSDPQQF | MGN GAR GCN NWN SMN ATG GAY CCN CAR CAN MG | general KS-specific primer | Fisch et al. 2009 |
| KF0004=  KSHGTGR | GGR TCN CCN ARN SWN GTN CCN GTN CCR TG | general KS-specific primer | Fisch et al. 2009 |

**Supplementary Table 2:** Isolated strains and their source

| Source | Closest relative strain | Internal Strain nr. |
| --- | --- | --- |
| *Chromodoris annae* (Chan15Bu-2) | Vibrio coralliilyticus strain OCN014 | Bu15_01 |
|  | Vibrio harveyi strain ATCC 33843 | Bu15_02 |
|  | Vibrio harveyi strain ATCC 33843 | Bu15_03 |
|  | Vibrio coralliilyticus strain OCN014 | Bu15_04 |
| *Chromodoris dianae* (Chdi15Bu-3 + Chdi15Bu-38 + Chdi15Bu-55) | Bacillus subtilis strain EDR4 | Bu15_05 |
|  | Serratia marcescens strain SW2-9-3 | Bu15_06 |
|  | Pseudovibrio sp. FO-BEG1 strain FO-BEG1 | Bu15_07 |
|  | Pseudoalteromonas sp. CF6-1 (+uncultured organism clone) | Bu15_08 |
|  | Pseudoalteromonas sp. AS-43 | Bu15_09 |
|  | Vibrio harveyi strain ATCC 3384 | Bu15_10 |
|  | Pseudovibrio sp. FO-BEG1 strain FO-BEG1 | Bu15_11 |
| *Chromodoris sp. 30* (Chsp3015Bu-4) | Vibrio harveyi strain ATCC 33843 | Bu15_12 |
|  | Pseudovibrio sp. FO-BEG1 | Bu15_13 |
|  | Vibrio harveyi strain ATCC 33843 | Bu15_14 |
| *Chromodoris williani* (Chwi15Bu-2) | Vibrio tubiashii ATCC 19109 | Bu15_15 |
| *Chromodoris annae eggmass* (Chan15Bu-11E) | Bacillus aryabhattai isolate PSB57 | Bu15_16 |
|  | Bacillus thuringiensis serovar indiana strain HD521 | Bu15_17 |
|  | Pseudoalteromonas sp. BJ9 | Bu15_18 |
|  | Pseudoalteromonas sp. BJ9 | Bu15_19 |
|  | Marinomonas communis strain NBRC 102224 (+uncultured organism clone) | Bu15_20 |
|  | Pseudovibrio sp. FO-BEG1 | Bu15_21 |
| *Doriprismatica stellata* (Glst15Bu-1) | Providencia vermicola strain FFA6 | Bu15_38 |
| *Doriprismatica stellata* eggmass (Glst15Bu-1E) | Vibrio alginolyticus NBRC 15630 | Bu15_32 |
|  | Vibrio alginolyticus NBRC 15630 | Bu15_33 |
|  | Vibrio alginolyticus NBRC 15630 | Bu15_34 |
|  | Pseudoalteromonas sp. AS-43 | Bu15_35 |
|  | Pseudoalteromonas sp. BJ9 (+uncultured organism clone) | Bu15_36 |
| *Doriprismatica stellata* sponge (Glst15Bu-1P) | Vibrio alginolyticus strain RE98 | Bu15_37 |
| *Hexabranchus sanguineus* eggmass (Hesa15Bu-1) | Longispora albida strain K97-0003 | Bu15_22 |
|  | Marinobacter sp. L21-PYE-C23 | Bu15_23 |
|  | Pseudoalteromonas sp. AS-43 | Bu15_24 |
|  | Pseudoalteromonas sp. AS-43 | Bu15_25 |
|  | Pseudoalteromonas sp. AS-43 | Bu15_26 |
|  | Vibrio alginolyticus NBRC 15630 | Bu15_27 |
|  | Vibrio sp. Ex25 | Bu15_28 |
|  | Bacillus thuringiensis serovar indiana strain HD521 | Bu15_29 |
|  | Vibrio alginolyticus NBRC 15630 | Bu15_30 |
|  | Pseudoalteromonas rubra | Bu15_31 |
| *Hexabranchus sanguineus* eggmass  (Hesa15Bu-2) | Pseudomonas sp. PETBA | Bu15_40 |
|  | Pseudomonas sp. MBEA06 | Bu15_47 |
|  | Microbacterium testaceum | Bu15_48 |
|  | Pelagibaca bermudensis | Bu15_49 |
|  | Microbacterium sp. SKJH-23 | Bu15_41 |
|  | Microbacterium sp. LHR-08 | Bu15_42 |
|  | Pseudomonas pseudoalcaligenes | Bu15_43 |
|  | Gordonia sp. VCM12 | Bu15_44 |
|  | Gordonia terrae | Bu15_45 |
|  | Pseudomonas pachastrellae | Bu15_46 |
| *Phyllidiella* cf *pustulosa* (Phpu15Bu-1) | Vibrio sp. BWDY-57 (+uncultured organism clone) | Bu15_39 |

# References

Ayuso-Sacido, A.and Genilloud, O. (2005): New PCR primers for the screening of NRPS and PKS-I systems in actinomycetes: detection and distribution of these biosynthetic gene sequences in major taxonomic groups. In: *Microbial ecology* 49 (1), S. 10–24. DOI: 10.1007/s00248-004-0249-6.

Schirmer, Andreas; Gadkari, Rishali; Reeves, Christopher D.; Ibrahim, Fadia; DeLong, Edward F.; Hutchinson, C. Richard (2005): Metagenomic analysis reveals diverse polyketide synthase gene clusters in microorganisms associated with the marine sponge Discodermia dissoluta. In: *Applied and environmental microbiology* 71 (8), S. 4840–4849. DOI: 10.1128/AEM.71.8.4840-4849.2005.

Wu, Xue-Chang; Qian, Chao-Dong; Fang, Hai-Huan; Wen, Yan-Ping; Zhou, Jian-Ying; Zhan, Zha-Jun et al. (2011): Paenimacrolidin, a novel macrolide antibiotic from Paenibacillus sp. F6-B70 active against methicillin-resistant Staphylococcus aureus. In: Microbial biotechnology 4 (4), S. 491–502. DOI: 10.1111/j.1751-7915.2010.00201.x.
